# LDL retention time in plasma can be -based on causation- estimated by the lipid composition of LDL and other lipoproteins

**DOI:** 10.1371/journal.pone.0272050

**Published:** 2022-07-28

**Authors:** Martin Jansen, Christine Contini

**Affiliations:** 1 Institute of Clinical Chemistry and Laboratory Medicine, Medical Centre -University of Freiburg, Freiburg im Breisgau, Germany; 2 Faculty of Medicine, University of Freiburg, Freiburg im Breisgau, Germany; China Medical University, TAIWAN

## Abstract

**Introduction:**

Information on LDL’s dynamic behaviour of LDL (i.e. production rate and fractional catabolic rate) are of interest if pathologies, lipid-lowering strategies or LDL-metabolism itself are investigated. Determination of these rates is costly and elaborate. Here we studied the interrelationship of LDL mass, its composition and other lipoproteins. Based on this data, we deducted information about LDL’s dynamic behaviour.

**Methods:**

Lipoprotein profiles of n = 236 participants are evaluated. Plasma was separated by sequential ultracentrifugation into VLDL, IDL, LDL and HDL. Additionally, LDL and HDL were separated into subfractions. Stepwise multiple linear regressions were used to study LDL’s ApoB mass and lipid composition. Relying on these results and on causation, we constructed a mathematical model to estimate LDL’s retention time.

**Results:**

The ApoB mass in LDL correlated best among all measured parameters (including corresponding lipid compositions but using no LDL-associated parameters) with the cholesterol ester content in IDL. TG/CE ratios in LDL’s subfractions were strongly correlated with the corresponding ratios in IDL and HDL. The constructed mathematical model links the TG/CE ratio of LDL and HDL to LDL’s ApoB concentration and enables a good estimate of LDL’s retention time in plasma.

**Discussion:**

Relying on our statistic evaluations, we assume that i) the production of nascent LDL via IDL as well as ii) LDL’s prolonged retention are mapped by the TG/CE ratio in LDL subfractions. HDL’s TG/CE ratio is associated with the change in LDL’s TG/CE ratio during its retention in plasma. Our mathematical model uses this information and enables–by relying on causation- a good estimation of LDL’s retention time.

## Introduction

One of the main tasks of lipoproteins is to mediate the distribution of lipids among the liver and peripheral tissues. In the fasting situation, lipoproteins are classified by their density into very low-, intermediate-, low- and high-density-lipoproteins (VLDL, IDL, LDL and HDL).

There is a strong association between lipoproteins–especially LDL- and cardiovascular risk [1]. To quantify LDL in plasma, parameters such as its cholesterol-, Apolipoprotein B-100- or particle-concentrations are determined. Further, LDL’s and HDL’s TG/CE ratio are associated with hyperlipidemia [2–4] and LDL’s composition and phenotype are associated to cardiovascular risk [5, 6].

Kinetic studies are necessary to access information about LDL’s dynamics, i.e. its production rate and fractional catabolic rate (FCR) [7]. Kinetic data is beneficial, if interventions to lower LDL-cholesterol are interpreted and evaluated. LDL mass depends on both the production and clearance rate of LDL, which is mostly mediated by a receptor- and to less extent by a receptor-independent-pathway [8]. In severe hypertriglyceridemia LDL clearance is increased due to stimulation of the receptor-independent-pathway [9]. Hence, kinetic measurements are necessary to clarify whether an elevated concentration of LDL-particles is caused by an impaired production- or clearance-rate. These measurements are highly elaborate in their experimental design (several time points, a bolus injection of isotopic-marked amino acids) and evaluation (determining tracer-tracee ratios via mass spectrography).

LDL’s neutral fats (triglycerides (TG) and cholesterol ester (CE)) are located in its hydrophobic core. During LDL’s retention in plasma, its CE amount is affected by Cholesterol Ester Transfer Protein (CETP) [10] and its TG amount is affected by CETP and hepatic lipase (HL) [11]. Furthermore, as LDL originates mostly from IDL [12], nascent LDL inherits its CE-load from IDL, and nascent LDL particles can enter all LDL-subfractions directly [13]. Combining those facts, we assume that is possible to obtain kinetic information about LDL by just considering the CE and TG composition of LDL, IDL and other lipoproteins.

## Methods

### Subjects

We used data from individuals, who can or cannot be allocated to a hyper- or hypolipoproteinaemia [14]. All subjects’ data were retrieved from previous studies [15], ongoing clinical studies, or from volunteers [16] and are approved by the Freiburg Ethics Committee. Each participant gave written consent. The study subjects were divided into a normolipidemic (NL), hypertriglyceridemic (HTG) or hypercholesteridemic (HCH) group based on their plasma TG and LDL-cholesterol as described elsewhere [15].

### Experimental procedure

Lipoproteins were separated and lipids and apolipoproteins then measured as described previously [16, 17]. In short, plasma was separated via sequential ultracentrifugation into VLDL, IDL, LDL and HDL. Additionally, LDL was separated into six (LDL1-LDL6, ranging from 1.019–1.031, 1.031–1.034, 1.034–1.037, 1.040–1.044 and 1.044–1.063 g/ml, respectively) and HDL into three (HDL2b, HDL2a, HDL3) subfractions [18]. Free cholesterol (FC), CE, phospholipids (PL) and TG were measured in all fractions. Apolipoproteins A1 (ApoA1), A2 (ApoA2), B-100 (ApoB) and Lipoprotein(a) (Lp(a)) were measured in the fractions and subfractions, where a significant concentration would be expected. As described in our previous work [16], TG, FC, total cholesterol and PL were determined enzymatically via PAP-methods, and the apolipoproteins were determined applying turbidimetric methods.

### Notations

Let *LP*_*x*_ and $LP_{\frac{y}{z}}$ denote the concentration of *x* and the molar *y/z* ratio in the lipoprotein fraction *LP*, respectively. *x*, *y* and *z* can either be a lipid (CE, FC, PL or TG) or an apolipoprotein (ApoA1 or ApoB).

### Statistics

We performed stepwise multiple linear regression analyses with a significance threshold of p<0.05 to asses variables associated to i) *LDL*_*ApoB*_ and ii) $LDL1_{\frac{TG}{CE}}\;-\;LDL6_{\frac{TG}{CE}}$. The fit of the linear model was assessed using the coefficient of determination R^2^.

We include all measured concentrations as well as lipid-to-ApoB ratios in all ApoB carrying lipoproteins and TG/CE ratios in all lipoprotein fractions. To omit redundancy, we excluded ApoB and lipid concentrations of LDL and total plasma (as plasma usually consists to a great extent of LDL mass) from our analysis in case i). We also excluded the TG/CE ratios in LDL and its subfractions in case ii). We checked the distributions of all parameters before including them to avoid the usage of highly non-Gaussian distributions in the linear regression. In detail, we log-transformed variables which were tested as non-Gaussian distributed (using Kolmogorov-Smirnov test, p<0.005). Associations between parameters were examined via Spearman’s rank correlation.

Differences between groups were analysed using the Mann-Whitney-U Test.

All statistical analyses were performed using IBM SPSS version 26 (IBM SPSS Statistics; IBM Corporation, Chicago, IL).

### Mathematical model

We constructed (relying on our statistic results) a mathematical model which links the retention time of LDL to LDL’s lipid composition and its underlying dynamics.

Let ${\bar{LDL}}_{\frac{TG}{CE}}\left(t\right)$ be a function displaying the mean molar TG/CE ratio of a nascent LDL particle with infinite plasma retention time *t* hours after its entrance in plasma. Its change in TG/CE is mediated by CETP and HL action. While CETP action enriches the TG poor LDL, HL action leads to a reduction in TG in LDL.

**Fig 1:**
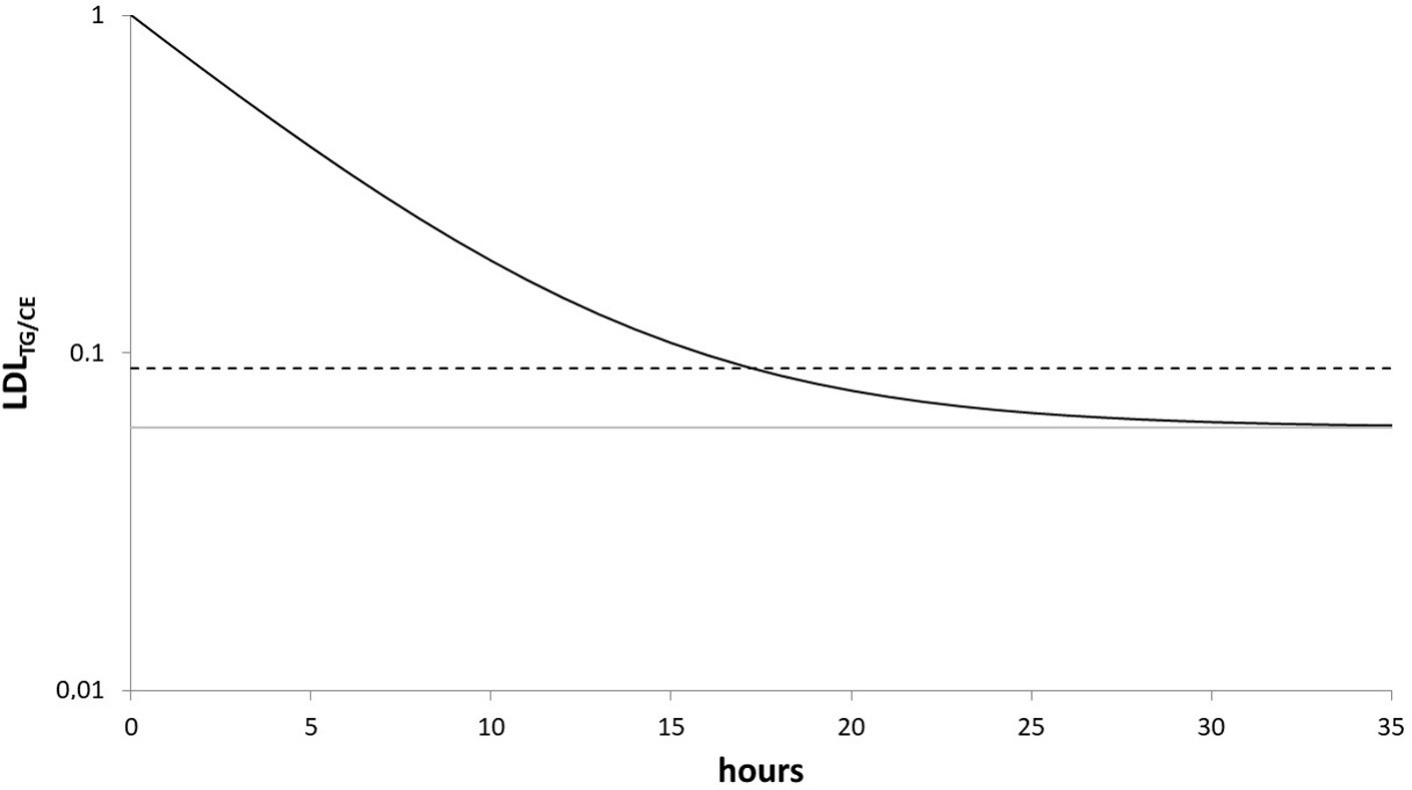
Sketch of the model. Black solid line: exemplary trajectory of the TG/CE ratio of a hypothetical LDL particle with infinite retention time in plasma $\left({\bar{\text{LDL}}}_{\frac{\text{TG}}{\text{CE}}}\left(\text{t}\right)\right)$. The ratio decreases exponentially with rate r. Grey line: The corresponding asymptote $\left({\text{Min}}_{\frac{\text{TG}}{\text{CE}}}\right)$. Dashed line: the observed molar TG/CE ratio in LDL $\left({\text{LDL}}_{\frac{\text{TG}}{\text{CE}}}\right)$, which is the mean of the TG/CE ratio of all present LDL particles.

Let’s assume i) ${\bar{LDL}}_{\frac{TG}{CE}}\left(0\right)=1$, hence that a nascent LDL particle has a TG/CE ratio of 1 and ii) $\underset{t\to \infty }{\text{lim}}{\bar{LDL}}_{\frac{TG}{CE}}\left(t\right)=Min_{\frac{TG}{CE}}$, hence the particle’s TG/CE ratio approximates an equilibrium $Min_{\frac{TG}{CE}}\leq 1$. Based on previous work, in which the underlying reactions and their properties are described and discussed [15, 17, 19] and in which experiments were performed to [15] to study the influence of CETP and LCAT on LDL’s TG and CE content, we iii) assume that the ratio ${\bar{LDL}}_{\frac{TG}{CE}}$ decreases exponentially during its retention. Let *r* be the corresponding reaction rate. This is of course a simplification, but as the true underlying reaction kinetics are not fully understood and complex (CETP seems to exchange TG between lipoproteins diffusion-like), we think modelling it as a first-order reaction is a potentially good approximation–also because, being the most natural choice, it does not increase the complexity in line with the principle of parsimony. Fig 1 visualises these assumptions.

Given assumptions i)-iii), differential equations calculus leads to the explicit representation:

$${\bar{LDL}}_{\frac{TG}{CE}}\left(t\right)=Min_{\frac{TG}{CE}}+\left(1-Min_{\frac{TG}{CE}}\right)\exp\left(-rt\right)$$ (Eq 1)

Note, that the probability density function F with rate parameter μ of an exponential distribution is defined as *F*(*t*;μ) = *μ*∙exp(−*μt*), if t≥0 and *F*(*t*;μ) = 0, elsewise. Let *μ* (in pools per days) be the rate for the exponentially distributed clearance of LDL particles from the blood. μ represents LDL’s ApoB FCR. The observed molar ratio $LDL_{\frac{TG}{CE}}$, which represents by definition the expected value of ${\bar{LDL}}_{\frac{TG}{CE}}\left(t\right)$, can be estimated as:

$$LDL_{\frac{TG}{CE}}=\overset{\infty }{\underset{0}{\int }}{\bar{LDL}}_{\frac{TG}{CE}}\left(t\right)\cdot F\left(t;\mu \right)dt$$ (Eq 2)

Combining (Eq 1) and (Eq 2), it holds:

$$\begin{array}{ccc} LDL_{\frac{TG}{CE}} & = & \overset{\infty }{\underset{0}{\int }}{\bar{LDL}}_{\frac{TG}{CE}}\left(t\right)\cdot \mu \cdot \exp\left(-\mu t\right)dt\\ & = & \mu \cdot \overset{\infty }{\underset{0}{\int }}\left(Min_{\frac{TG}{CE}}+\left(1-Min_{\frac{TG}{CE}}\right)\exp\left(-rt\right)\right)\cdot \exp\left(-\mu t\right)dt\\ & = & Min_{\frac{TG}{CE}}+\left(1-Min_{\frac{TG}{CE}}\right)\frac{\mu }{\mu +r} \end{array}$$ (Eq 3)

Eventually it follows:

$$\frac{\mu }{\mu +r}=\frac{LDL_{\frac{TG}{CE}}-Min_{\frac{TG}{CE}}}{1-Min_{\frac{TG}{CE}}}$$ (Eq 4)

So instead of the desired rate *μ*, which represents LDL’s ApoB FCR, we have derived a term describing the ratio $\frac{\mu }{\mu +r}$. We have no direct information about rate *r* (the decrease of the TG/CE ratio in a LDL particle mediated by HL and CETP action). However, given *r* is independent from *μ* and has low variance, the derived ratio is strongly associated (non-linearly) with *μ*. This assumption is supported by Jansen et al., who state that HL’s influence on LDL’s phenotype in non-hypertriglyceridemic conditions is rather weak [20].

### Estimating LDL’s ApoB-FCR

Considering our data, w we have no direct information about the *LDL*_*ApoB*_-FCR. We want to use the best surrogate parameter available by taking non-kinetic measurements. Considering the literature and our own intuition, LDL-cholesterol and *LDL*_*ApoB*_ fulfil this requirement as they correlate well with the FCR [21–24]. Packard et al report a linear correlation of *r* = −0.77 (*P* < 0.001, n = 20) [21]. This correlation relies on causation as assuming a constant production of LDL, it holds:

$$LDL_{ApoB}=\frac{Production\;of\;LDL}{LDL_{ApoB}\text{FCR}}$$ (Eq 5)

Hence, if variability in the *LDL*_*ApoB*_-FCR rather than in production is responsible for the quantity of ApoB in LDL, *LDL*_*ApoB*_-FCR and $\frac{1}{LDL_{ApoB}}$ are strongly linearly correlated. Thus we checked the (non-parametrical) correlation between the FCR-associated parameter derived from our model $\frac{\mu }{\mu +r}$ and the *LDL*_*ApoB*_-FCR estimator $\frac{1}{LDL_{ApoB}}$ derived from our data and described in this section in an additional analysis.

## Results

**Fig 2:**
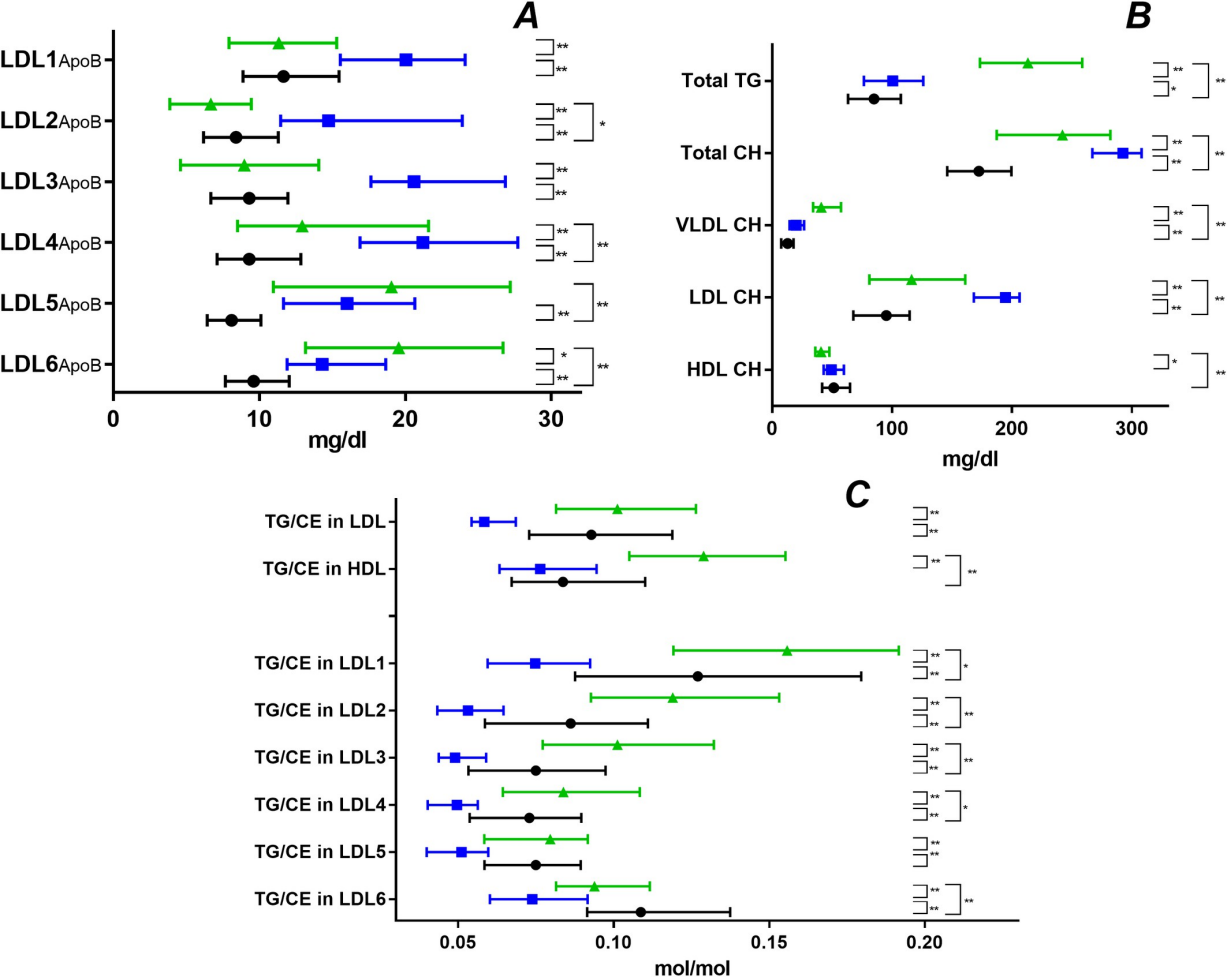
Lipoprotein characteristics. Quartiles in NL (black), HTG (green) and HCH (blue). (A) ApoB in the LDL subfractions, (B) CH and TG in total and CH in lipoprotein fractions (C) TG/CE ratios in HDL, LDL and its subfractions. Statistical significance determined by Mann-Whitney-U test (*p < 0.05, **p < 0.01).

n = 236 individuals were included in this analysis, data on n = 91 subjects were already used in a prior study [15]. 145, 27 and 64 individuals were classified as NL, HTG and HCH, respectively (Fig 2).

### Regression analysis of *LDL*_*ApoB*_ and the ratios $LDL1_{\frac{TG}{CE}}$ − $LDL6_{\frac{TG}{CE}}$

We performed linear stepwise multiple regression to seek the best parameters explaining *LDL*_*ApoB*_ and the ratios $LDL1_{\frac{TG}{CE}}-LDL6_{\frac{TG}{CE}}$ in a linear model. In Tables 1 and 2, we displayed only the two best explanatory variables. In all our results, the first is simultaneously the best linear correlating variable and -adjusted for it- the second explanatory variable is the best linear correlating parameter. The following parameters were log-transformed, when used in linear regressions: *IDL*_*TG*_, *IDL*_*CE*_,*VLDL*_*TG*_, $IDL_{\frac{TG}{ApoB}}$, $IDL_{\frac{TG}{CE}},\;HDL2b_{CE},\;HDL2b_{TG}$, $LDL_{\frac{TG}{CE}}$, $LDL_{\frac{CE}{ApoB}}$. The parameters LP(a) and $HDL2b_{\frac{CE}{ApoA1}}$ were excluded from our analysis.

**Table 1:** Stepwise linear multiple regression on ApoB in LDL.

	explanatory variable 1	R^2^	explanatory variable 2	R^2^
All (n = 236)	*IDL*_*CE*_	0.508	$LDL_{\frac{TG}{ApoB}}$	0.621
Normolipidemic (n = 145)	*IDL*_*CE*_	0.353	$LDL_{\frac{TG}{ApoB}}$	0.534
Hypercholesterolemia (n = 27)	$IDL_{\frac{PL}{ApoB}}$	0.393	-	
Hypertriglyceridemia (n = 64)	*IDL*_*CE*_	0.570	$LDL_{\frac{TG}{ApoB}}$	0.646

Stepwise multiple linear regression analysis, dependent variable:

*LDL*_*ApoB*_ (ApoB in LDL). The best two explanatory variables (if existing) are illustrated. All lipid and apolipoprotein concentrations and calculated compositions in all measured lipoprotein fractions, but LDL and its subfractions were included in the analysis. It holds in all cases: p<0.001. R^2^; coefficient of determination.

**Table 2:** Linear multiple regression on lipid composition in LDL’s subfractions.

		$LDL1_{\frac{TG}{CE}}$		$LDL2_{\frac{TG}{CE}}$		$LDL3_{\frac{TG}{CE}}$		$LDL4_{\frac{TG}{CE}}$		$LDL5_{\frac{TG}{CE}}$		$LDL6_{\frac{TG}{CE}}$	
		explanatory variable	R^2^	explanatory variable	R^2^	explanatory variable	R^2^	explanatory variable	R^2^	explanatory variable	R^2^	explanatory variable	R^2^
Total (n = 236)	1	$IDL_{\frac{TG}{CE}}$	0.320	$HDL2b_{\frac{TG}{CE}}$	0.468	$HDL2b_{\frac{TG}{CE}}$	0.456	$HDL2b_{\frac{TG}{CE}}$	0.371	$IDL_{\frac{TG}{CE}}$	0.292	$IDL_{\frac{TG}{CE}}$	0.406
	2	$HDL2b_{\frac{TG}{CE}}$	0.569	$IDL_{\frac{TG}{CE}}$	0.613	$IDL_{\frac{TG}{CE}}$	0.588	$IDL_{\frac{TG}{CE}}$	0.545	$HDL_{\frac{TG}{CE}}$	0.564	$HDL_{\frac{TG}{CE}}$	0.540
Normolipidemic (n = 145)	1	$IDL_{\frac{TG}{CE}}$	0.375	$IDL_{\frac{CE}{TG}}$	0.296	$IDL_{\frac{TG}{CE}}$	0.334	$IDL_{\frac{TG}{CE}}$	0.391	$IDL_{\frac{TG}{CE}}$	0.436	$IDL_{\frac{TG}{CE}}$	0.425
	2	$HDL2b_{\frac{TG}{CE}}$	0.520	$HDL2b_{\frac{TG}{CE}}$	0.518	$HDL2b_{\frac{TG}{CE}}$	0.513	*HDL*2*b*_*TG*_	0.462	*HDL*2*b*_*TG*_	0.513	$HDL2b_{\frac{TG}{CE}}$	0.537
Hypercholesterolemia (n = 27)	1	$HDL2b_{\frac{TG}{CE}}$	0.360	$HDL2b_{\frac{TG}{CE}}$	0.663	$HDL2b_{\frac{TG}{CE}}$	0.790	$HDL2b_{\frac{TG}{CE}}$	0.699	$HDL2b_{\frac{TG}{CE}}$	0.510	*IDL*_*CE*_	0.410
	2	$HDL2a_{\frac{TG}{CE}}$	0.757	-		-		*IDL*_*CE*_	0.769	*IDL*_*CE*_	0.697	$HDL2b_{\frac{TG}{CE}}$	0.651
Hypertriglyceridemia (n = 64)	1	$HDL2b_{\frac{TG}{CE}}$	0.542	$HDL2b_{\frac{TG}{CE}}$	0.677	$HDL2b_{\frac{TG}{CE}}$	0.618	$HDL2b_{\frac{TG}{CE}}$	0.677	$HDL_{\frac{TG}{CE}}$	0.790	$HDL_{\frac{TG}{CE}}$	0.523
	2	$IDL_{\frac{TG}{CE}}$	0.696	-		*IDL*_*TG*_	0.645	*VLDL*_*ApoB*_	0.753	*HDL*2*a*_*TG*_	0.805	*IDL*_*CE*_	0.567

Stepwise multiple linear regression analysis, dependent variables are the molar TG to CE ratios in the LDL subfractions $LDL1_{\frac{TG}{CE}}\;–LDL6_{\frac{TG}{CE}}$, repectively. The best two explanatory variables (if existing) are displayed. All lipid and apolipoprotein concentrations and calculated compositions in all measured lipoprotein fractions but TG to CE ratios in the total LDL fractions and its subfractions were included in the analysis, It holds in all cases: p<0.001. R^2^, coefficient of determination.

1. *LDL*_*ApoB*_ modelled with linear regression:

The best two explanatory variables of the regression were *IDL*_*CE*_ and $LDL_{\frac{TG}{ApoB}}$ (Table 1). Considering the three different subpopulations, *IDL*_*CE*_ was the first explanatory variable in all but the HCH case.

1. The ratios $LDL1_{\frac{TG}{CE}}-LDL6_{\frac{TG}{CE}}$ modelled with linear regression:
2. $IDL_{\frac{TG}{CE}}$ and $HDL2b_{\frac{TG}{CE}}$ were the two most important explanatory variables in the regression (Table 2). $IDL_{\frac{TG}{CE}}$ was the best explanatory variable for $LDL1_{\frac{TG}{CE}}-LDL6_{\frac{TG}{CE}}$ in the NL situation (in all cases). In the HCH situation $HDL2b_{\frac{TG}{CE}}$ is the best explanatory variable for $LDL1_{\frac{TG}{CE}}-LDL5_{\frac{TG}{CE}}$, while *IDL*_*CE*_ is the best explanatory variable for $LDL6_{\frac{TG}{CE}}$. In the HTG-case $HDL2b_{\frac{TG}{CE}}$ and $HDL_{\frac{TG}{CE}}$ were the best explanatory variables for $LDL1_{\frac{TG}{CE}}-LDL4_{\frac{TG}{CE}}$ and $LDL5_{\frac{TG}{CE}}-LDL6_{\frac{TG}{CE}}$, respectively.

**Fig 3:**
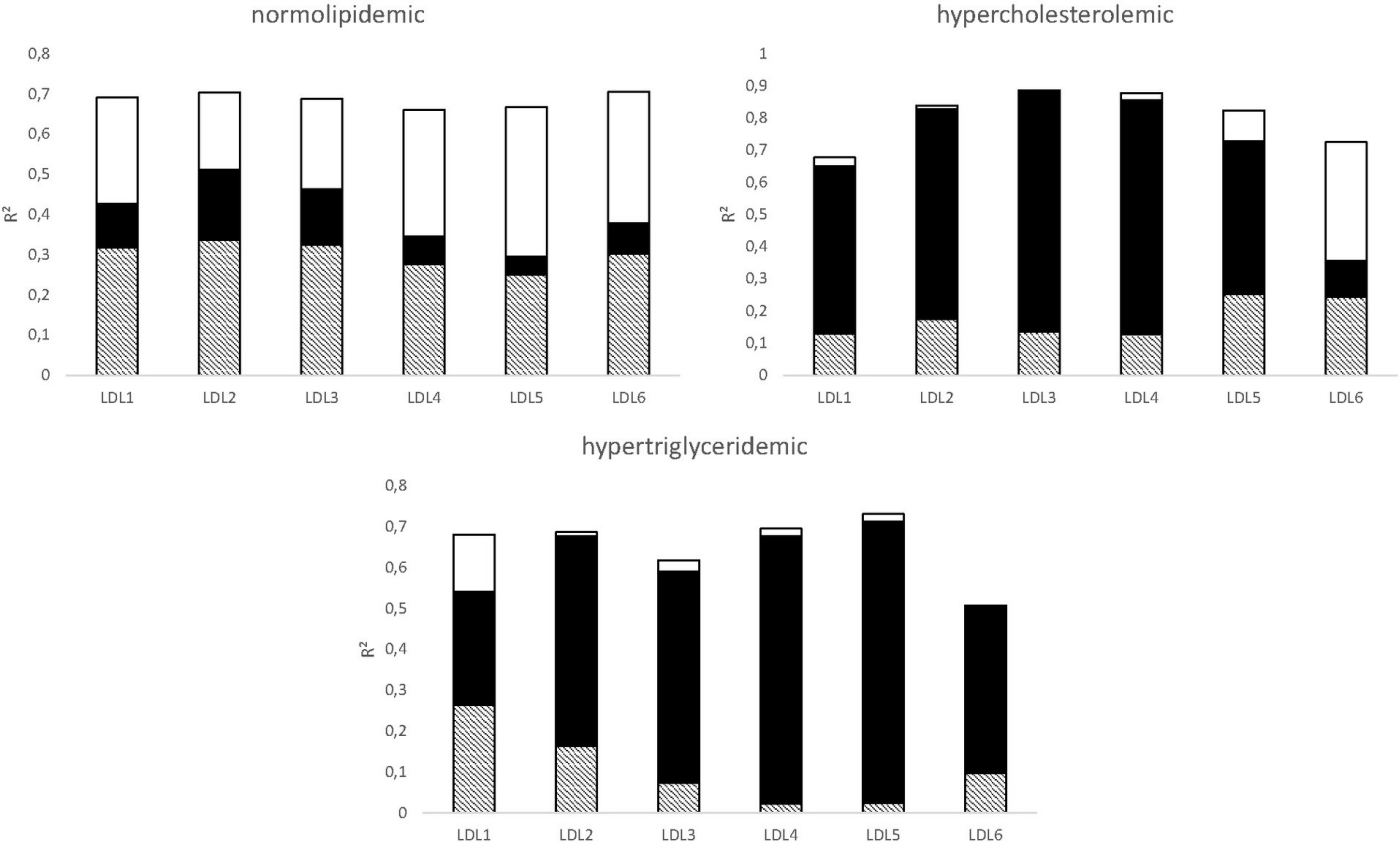
Estimating the influence of CETP on LDL’s lipid composition. The bar represents the coefficient of determination R^2^ of a multiple linear regression model predicting $LDL1_{\frac{TG}{CE}}-LDL6_{\frac{TG}{CE}}$ using the two explanatory variables $V1:IDL_{\frac{TG}{CE}}$ and V2: $HDL2b_{\frac{TG}{CE}}$ in 3 subgroups. Let r^2^(*V*1) and r^2^(*V*2) be the coefficients of determination, if only *V*1 or *V*2 are used as explanatory variable in a corresponding single linear regression, respectively. Three parts contribute to R^2^: V1 without V2 (white, R^2^-r^2^(*V*2)), V2 without V1 (black, R^2^-r^2^(*V*2)), and V1 and V2 intersecting (dashed, r^2^(*V*1)+ r^2^(*V*2)-R^2^).

Fig 3 displays to what degree the ratios $LDL1_{\frac{TG}{CE}}-LDL6_{\frac{TG}{CE}}$ can be described by a linear model given the two explanatory variable $HDL2b_{\frac{TG}{CE}}$ and $IDL_{\frac{TG}{CE}}$.

### The mathematical model

Based on the results of the linear regressions and our previous research [15], we chose $Min_{\frac{TG}{CE}}$ to be proportional to $HDL_{\frac{TG}{CE}}$, as we deduced that $HDL_{\frac{TG}{CE}}$ is associated with the TG/CE ratio’s equilibrium given CETP and HL action. Note that in more than 60% of our subjects it holds $HDL_{\frac{TG}{CE}}>\;LDL_{\frac{TG}{CE}}$. Hence, to ensure that $Min_{\frac{TG}{CE}}$ is lower than the lowest TG/CE ratio in LDL (which it should be by definition), we scaled $HDL_{\frac{TG}{CE}}$ by 0.6. We chose 0.6, as $HDL_{\frac{TG}{CE}}*0.6<\;LDL_{\frac{TG}{CE}}$ was detected in 95% of our subjects. Consequently, it holds following (Eq 4):

$$\frac{\mu }{\mu +r}=\frac{LDL_{\frac{TG}{CE}}-\;HDL_{\frac{TG}{CE}}*0.6}{1-\;HDL_{\frac{TG}{CE}}*0.6}$$ (Eq 6)

**Fig 4:**
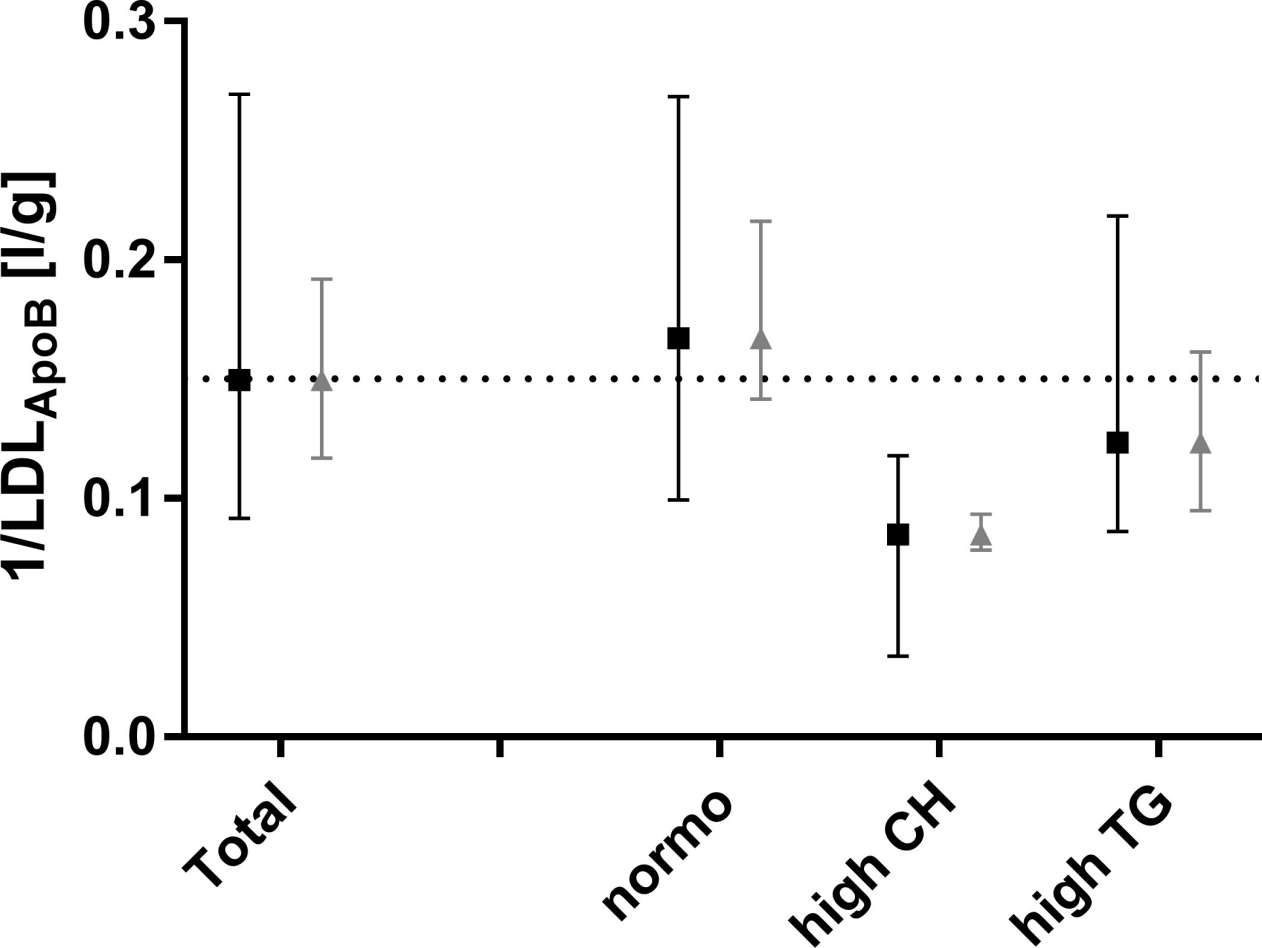
LDL-FCR estimation. Quartiles of the FCR estimator 1/*LDL*_*ApoB*_ (grey) and the model parameter $\frac{\mu }{\mu +r}$ (black). The model parameter is normalised to the median (dotted line) of 1/*LDL*_*ApoB*_ in ‘Total’).

Table 3 displays correlations for the FCR-estimator $\left(\frac{1}{LDL_{ApoB}}\right)$. Parameters which correlated obviously with *LDL*_*ApoB*_ (by name: CE, FC, PL and ApoB in total plasma and LDL), were excluded. The calculated parameter ratio $\frac{\mu }{\mu +r}$ correlates best with the FCR-estimator in the HCH case. In the NL case, it is the second best correlating variable after *IDL*_*CE*_. Only in the HTG case did *IDL*_*CE*_ and other IDL-parameters correlate clearly better than $\frac{\mu }{\mu +r}$ with the FCR-estimator. Fig 4 compares $\frac{1}{LDL_{ApoB}}$ and $\frac{\mu }{\mu +r}$ between all subjects and the three subgroups. Both parameter’s medians are similar, however the model parameters interquartile range is distinctly broader than the FCR-estimator’s.

**Table 3:** Comparison of correlations associated with 1/*LDL*_*ApoB*_.

	*IDL*_*CE*_	$\frac{\mu }{\mu +r}$	$LDL_{\frac{TG}{CE}}$	$HDL_{\frac{TG}{CE}}$	$LDL_{\frac{TG}{ApoB}}$
All (n = 236)	-0.689**	0.667**	0.474**	-0.105	0.432**
NL (n = 145)	-0.586**	0.581**	0.437**	-0.131	0.383**
HCH (n = 27)	-0.422*	0.617**	0.161	-0.287	0.233
HTG (n = 64)	-0.735**	0.487**	0.458*	0.210	0.386**

Non-parametric correlation (Spearman’ rho) between 1/*LDL*_*ApoB*_ (which acts as FCR estimator) and the following parameters: *IDL*_*CE*_(the best scoring non-LDL parameter), $LDL_{\frac{TG}{ApoB}}$ (derived from linear regression, Table 1), the model parameter $\frac{\mu }{\mu +r}\;,\;LDL_{\frac{TG}{CE}}$ and $HDL_{\frac{TG}{CE}}$ (as they are part of the term $\frac{\mu }{\mu +r}$).

* p<0.05,

**p<0.001

## Discussion

The heterogeneity of the lipid profiles in our analyses covers the normal NL situation as well as the two important pathological conditions hypertriglyceridemia and hypercholesterolemia. The data used in our analyses has high resolution as not only VLDL, LDL and HDL but IDL and LDL’s and HDL’s subfractions are isolated and as besides classic lipids and apolipoproteins (such as cholesterol, ApoA1 and ApoB) we additionally measured FC, PL and ApoA2. Hence, together with the relatively high number of samples (n = 236) this data is a solid base for our analyses.

The associations described by our regression analyses (Tables 1 and 2) allow us to formulate hypotheses about causal relationships between LDL’s mass and composition and other lipoprotein-data.

### LDL_ApoB_

The results of the linear regression (Table 1) suggest that (in all cases but HCH) the ApoB mass of LDL particles depends strongly on *IDL*_*CE*_. This is not surprising, as IDL is the main source of LDL [12]. *IDL*_*CE*_ might be superior to other IDL parameters like *IDL*_*ApoB*_, as it holds information as well about IDL’s kinetic properties: A less efficient transformation of IDL to LDL leads to IDL’s longer retention time, which itself leads to CE accumulation in IDL particles (via CETP-action). Hence, *IDL*_*CE*_ might contain information about both *IDL*_*ApoB*_-driven LDL production and IDL’s catabolic rate, which is coupled with LDL’s FCR.

The ratio $LDL_{\frac{TG}{ApoB}}$ is the second explanatory variable in the linear regression in all but the HCH case. Hence, adjusted to *IDL*_*CE*_ it contains additional information about LDL’s ApoB mass. It correlates negatively with *LDL*_*ApoB*_. This is in accordance with the hypothesis that LDL’s prolonged retention in plasma lowers its TG content [19]. In contrast to the model of van Schalkwijk et al. [19], we considered not only delipidation by lipases like HL but also the CETP-mediated net-flux of TG to LDL. This TG net-flux depends strongly on VLDL’s and HDL’s composition and concentration [15] and is incorporated within our model by letting LDL’s TG/CE ratio drop exponentially to an asymptote, which depends on CETP-mediated TG fluxes (Fig 1).

In the HCH-case, the production loses its significance as a factor influencing LDL’s mass in plasma, as the clearance via LDL-receptor is impaired. Furthermore, IDL’s production rate is significantly lower in HCH than in NL [25]. Thus considering our data, *IDL*_*CE*_ is not the correspondingly most significant explanatory variable, but rather $IDL_{\frac{PL}{ApoB}}$.

### The TG/CE ratio in LDL subfractions

Considering the explanatory variables of the linear regression in Table 2, there are two categories of variables, which are important: IDL-associated variables like $IDL_{\frac{TG}{CE}}$ and $IDL_{\frac{CE}{ApoB}}$ and HDL-associated variables like $HDL2b_{\frac{TG}{CE}}$ and $HDL_{\frac{TG}{CE}}$. The IDL-associated variables may be interpreted as the influence of LDL production and the HDL-associated variables may be interpreted as the influence of prolonged plasma retention on the $\frac{TG}{CE}$ ratio of LDL’s subfractions.

A linear combination of $HDL2b_{\frac{TG}{CE}}$ and $IDL_{\frac{TG}{CE}}$ holds a strong explanatory potential for the ratios $LDL1_{\frac{TG}{CE}}-\;LDL6_{\frac{TG}{CE}}$ (Fig 3). In the NL group, all LDL subfractions are strongly associated with IDL, which is in line with the assumption that LDL’s ApoB FCR is higher than in non-NL individuals. The subfractions in the HCH and HTG groups are strongly associated to $HDL2b_{\frac{TG}{CE}}$, which might be caused by the prolonged retention and stronger CETP-mediated TG exchange, respectively.

Considering the HCH group, LDL5 and LDL6 seem to be transient states, while LDL3 and LDL4 seem to form a sink, concurring with the observation that ApoB accumulates in FH in the middle dense LDL fractions [26]. In the HTG situation, LDL4 and LDL5 seem to form a sink. As HTG is associated with small, dense LDL, this observation also meets one’s expectation [27].

The strong association between the densest fraction $LDL6_{\frac{TG}{CE}}$ and production in all but the HTG-group is somehow surprising, as there is evidence that metabolically small dense LDL is derived out of buoyant LDL [21].

Boren et al. [28] presented a conceptual model describing the genesis and interrelationship of LDL subfractions dependent on the level of plasma TG. Consistent with our data (Fig 2) small, dense LDL accumulates if plasma TG is high. In contrast to the case where the TG level is normal or low, CETP action is important for the transformation of normal sized LDL to small, dense LDL, if plasma TG is high [28]. We also observed this trend in our analysis (Table 2 and Fig 3).

### Our mathematical model

Our model-derivation uses information obtained by studying the ApoB content and lipid-composition of LDL and its subfractions. Keeping in mind that the parameter $\frac{\mu }{\mu +r}$ is not derived out of IDL or LDL mass but out of the two compositional ratios $HDL_{\frac{TG}{CE}}$ and $LDL_{\frac{TG}{CE}}$ based on our understanding of how LDL metabolism works, the level of correlation between $\frac{\mu }{\mu +r}$ and the LDL-FCR estimator $\frac{1}{LDL_{ApoB}}$ (Table 3) is surprisingly high. The level of correlation is in all cases superior to the corresponding level of its components $HDL_{\frac{TG}{CE}}$ and $LDL_{\frac{TG}{CE}}$ (Eq 6).

A central assumption of our model is that there is a connection between HDL’s TG/CE and LDL’s TG/CE ratio: At steady state, both converge to an equilibrium due to CETP and HL action. In contrast to the relatively slow LDL metabolism, HDL reaches equilibrium in its TG/CE ratio very fast. This assumption is supported by our previous work, in which CETP’s dynamics are studied [15], and the linear regressions described in this manuscript. Therefore, it is reasonable to use HDL’s TG/CE ratio to define the corresponding equilibrium in LDL (Fig 1). This way we include HL and CETP action to our model.

The weak correlation between $\frac{\mu }{\mu +r}$ and the FCR-estimator $\frac{1}{LDL_{ApoB}}$ in the HTG subgroup (especially coinciding with extremely high serum TG) may be caused by

- the impact of the strongly altered TG-associated parameter *r*,
- extremely skewed LDL and HDL profiles (in composition and subfraction distribution) and the difficulty of separating HDL from LDL via ultracentrifugation—especially in individuals with very high total TG,
- altered LDL-catabolism in severe HTG [9] and
- the fact that we (in contrast to the NL and HCH groups) cannot expect our measurements to represent a quasi-stable state regarding TGs in LDL and HDL due to slower TG-catabolism.
- The receptor-independent-pathway clearance of LDL has a stronger impact on total LDL clearance [9]. If it exhibits a different kinetic behaviour than the receptor-mediated clearance, it violates our model-assumptions.

A clear limitation of our study is that we did not determine the real *LDL*_*ApoB*_-FCR, but used $\frac{1}{LDL_{ApoB}}$ as substitute-parameter. The estimated kinetic data derived by our model is of course clearly inferior compared to kinetic data derived by labelling molecules with stable isotopes to create tracers in vivo.

Our statistical results provide new insights on interdependencies among the mass of LDL’s subfractions and LDL’s lipid composition and other lipoproteins. We constructed, based on these results, a parameter, which creates a link between LDL’s lipid composition and its plasma-retention time. This link between lipoprotein composition and kinetic behaviour is in our view the most important aspect of this work. Our model may be employed for interpretation and evaluating of LDL-associated interventions, if no kinetic data is available.

## Supporting information

S1 Table Underlying data. This table holds all underlying data.
(XLSX)

S2 Table Non-parametric correlations of 1/*LDL*_*ApoB*_ and FCR-associated parameters. (DOCX)
