# Supplementary material for: LDL retention time in plasma can be -based on causation- estimated by the lipid composition of LDL and other lipoproteins
Source: PLoS One. 2022 Jul 28;17(7):e0272050. doi: 10.1371/journal.pone.0272050 (PMC9333322; doi:10.1371/journal.pone.0272050)
Supplement: S2 Table — (DOCX) [file pone.0272050.s002.docx]

Supplemental table 2: Non-parametric correlations of 1/${LDL}_{ApoB}$and FCR-associated parameters

**A**

|  | 1/${LDL}_{ApoB}$ | ${IDL}_{CE}$ | $\frac{\mu}{\mu+r}$ | ${LDL}_{\frac{TG}{CE}}$ | ${HDL}_{\frac{TG}{CE}}$ |
| --- | --- | --- | --- | --- | --- |
| ${IDL}_{CE}$ | 0,667^**^ |  |  |  |  |
| $\frac{\mu}{\mu+r}$ | -0,689^**^ | -0,495^**^ |  |  |  |
| ${LDL}_{\frac{TG}{CE}}$ | 0,474^**^ | 0,689^**^ | -0,128^*^ |  |  |
| ${HDL}_{\frac{TG}{CE}}$ | -0,105 | -0,099 | 0,431^**^ | 0,570^**^ |  |
| ${LDL}_{\frac{TG}{ApoB}}$ | 0,432^**^ | 0,653^**^ | -0,075 | 0,972^**^ | 0,573^**^ |

**B**

|  | 1/${LDL}_{ApoB}$ | ${IDL}_{CE}$ | $\frac{\mu}{\mu+r}$ | ${LDL}_{\frac{TG}{CE}}$ | ${HDL}_{\frac{TG}{CE}}$ |
| --- | --- | --- | --- | --- | --- |
| ${IDL}_{CE}$ | 0,581^**^ |  |  |  |  |
| $\frac{\mu}{\mu+r}$ | -0,586^**^ | -0,385^**^ |  |  |  |
| ${LDL}_{\frac{TG}{CE}}$ | 0,437^**^ | 0,760^**^ | -0,061 |  |  |
| ${HDL}_{\frac{TG}{CE}}$ | -0,131 | -0,106 | 0,492^**^ | 0,491^**^ |  |
| ${LDL}_{\frac{TG}{ApoB}}$ | 0,383^**^ | 0,711^**^ | 0,015 | 0,977^**^ | 0,524^**^ |

**C**

|  | 1/${LDL}_{ApoB}$ | ${IDL}_{CE}$ | $\frac{\mu}{\mu+r}$ | ${LDL}_{\frac{TG}{CE}}$ | ${HDL}_{\frac{TG}{CE}}$ |
| --- | --- | --- | --- | --- | --- |
| ${IDL}_{CE}$ | 0,617^**^ |  |  |  |  |
| $\frac{\mu}{\mu+r}$ | -0,422^*^ | -0,469^*^ |  |  |  |
| ${LDL}_{\frac{TG}{CE}}$ | 0,161 | 0,363 | -0,092 |  |  |
| ${HDL}_{\frac{TG}{CE}}$ | -0,287 | -0,437^*^ | 0,273 | 0,634^**^ |  |
| ${LDL}_{\frac{TG}{ApoB}}$ | 0,233 | 0,372 | -0,030 | 0,954^**^ | 0,576^**^ |

**D**

|  | 1/${LDL}_{ApoB}$ | ${IDL}_{CE}$ | $\frac{\mu}{\mu+r}$ | ${LDL}_{\frac{TG}{CE}}$ | ${HDL}_{\frac{TG}{CE}}$ |
| --- | --- | --- | --- | --- | --- |
| ${IDL}_{CE}$ | 0,487^**^ |  |  |  |  |
| $\frac{\mu}{\mu+r}$ | -0,735^**^ | -0,282^*^ |  |  |  |
| ${LDL}_{\frac{TG}{CE}}$ | 0,458^**^ | 0,670^**^ | -0,208 |  |  |
| ${HDL}_{\frac{TG}{CE}}$ | 0,210 | 0,187 | -0,051 | 0,809^**^ |  |
| ${LDL}_{\frac{TG}{ApoB}}$ | 0,386^**^ | 0,619^**^ | -0,115 | 0,943^**^ | 0,779^**^ |

Non-parametric correlations (Spearman’ rho) of 1/${LDL}_{ApoB}$ (which acts as FCR estimator), ${IDL}_{CE}$ (the best scoring non-LDL parameter), ${LDL}_{\frac{TG}{ApoB}}$ (derived from linear regression, Table 1), the model parameter$\frac{\mu}{\mu+r}$ , ${LDL}_{\frac{TG}{CE}}$ and${HDL}_{\frac{TG}{CE}}$ (as they are part of the term $\frac{\mu}{\mu+r}$). A: all cases (n=236), B: NL (n=145), C: HCH (n=27), D: HTG (n=64). * p<0.05, **p<0.001
